# Problematic Pornography Use in Japan: A Preliminary Study Among University Students

**DOI:** 10.3389/fpsyg.2021.638354

**Published:** 2021-04-16

**Authors:** Yushun Okabe, Fumito Takahashi, Daisuke Ito

**Affiliations:** ^1^Institute of Education, Shinshu University, Nagano, Japan; ^2^Graduate School of Education, Hyogo University of Teacher Education, Kato, Japan

**Keywords:** problematic pornography use, university students, Japan, addictive behavior, sexual compulsivity, depression, anxiety, effortful control

## Abstract

**Background:**

Problematic pornography use is considered an addictive behavior, which is an important clinical issue. Despite considerable research interest in problematic pornography use worldwide, to the best of our knowledge, there are no extant studies on the subject in Japan. Therefore, despite the fact that many people in Japan use pornography, the difference between problematic and non-problematic users among Japanese people is not known.

**Objective:**

This study aimed to identify the characteristics of problematic pornography use among Japanese students, to the best of our knowledge. Specifically, we examined general psychopathological symptoms, sexual compulsivity, depression, anxiety, and low effortful control.

**Methods:**

The participants were 150 college students aged 20–26 years (mean age = 21.5, *SD* = 1.21, males: *n* = 86, females: *n* = 64) at a university in midland Japan. An online questionnaire was administered that included items on pornography usage patterns, impaired control of pornography use, sexual compulsivity, depression, anxiety, and effortful control.

**Results:**

Most men (97%) and approximately one-third of women (35.9%) used pornography at least once in the past month. Some users reported significant daily-life problems due to difficulty in controlling pornography use (5.7%). Participants with impaired control of pornography use had higher depression, anxiety, and sexual compulsivity, and lower effortful control than pornography users without impaired control.

**Conclusion:**

Some Japanese students reported significant daily-life problems due to impaired control of pornography use. The characteristics of individuals with impaired control are consistent with previous studies. The results of this study suggest that individuals with impaired control may have poor mental health, and that there is need for further research and development of treatment systems to manage this issue in Japan. Further research exploring a more varied sample in Japan is required to effectively examine problematic pornography use.

## Introduction

Pornography use is an increasingly common behavior worldwide. Although many users have reported positive effects of pornography use (Hald and Malamuth, 2008), some have reported negative effects due to excessive use (Gola and Potenza, 2016). According to a recent review, many studies describe excessive pornography use as having a negative effect as a potential behavioral addiction, such as internet addiction (de Alarcón et al., 2019). Among researchers, it is referred to as problematic pornography use (Fernandez and Griffiths, 2019), and is characterized by control difficulties, excessive use, avoidance of negative emotions, and persistent use despite negative consequences (Kor et al., 2014). In addition, problematic pornography use could be interpreted in a behavioral addiction framework, which includes the six core components of addiction (Griffiths, 2005): salience (when an activity becomes the most important thing in one’s life), mood modification (using behavior to modify one’s mood state), withdrawal (an unpleasant emotional state that is experienced when the behavior is stopped), tolerance (needing an increase in behavior frequency to achieve similar effects), relapse (reverting to earlier behavior patterns after abstinence), and conflict (harmful consequences of the addictive behavior) (Fernandez and Griffiths, 2019; Chen and Jiang, 2020). Problematic pornography use is also related to other addictive behaviors, namely, hypersexuality, gambling, internet, and gaming (Kor et al., 2014; Stockdale and Coyne, 2018). Although problematic pornography use is known to have negative consequences as with other addictive behaviors, it has not been investigated in the context of Japan where the level of pornography use is high. This study expands upon the existing literature that focuses on problematic pornography use as a growing worldwide phenomenon by reporting the characteristics of problematic pornography use among Japanese students. Specifically, we examined general psychopathological symptoms, sexual compulsivity, depression, anxiety, and effortful control.

Given that problematic pornography use is considered a part of compulsive sexual behavior disorder under impulse-control disorders in the International Classification of Diseases 11th Revision (World Health Organization, 2018; Brand et al., 2019a), related research has received considerable attention in recent years (e.g., Kraus et al., 2020). Although not explicitly stated in the diagnosis, other sexual behaviors considered to be compulsive sexual behavior might include masturbation, telephone sex, cybersex, strip clubs, and sexual acts with consenting adults (Kafka, 2010; Gola et al., 2020). These sexual behaviors can be divided into two categories: individual-based, which do not require the involvement of a partner (e.g., masturbation); and partnered-based, which do require the involvement of a partner (e.g., repeated infidelity) (Efrati and Mikulincer, 2018). Problematic pornography use classified as an individual-based sexual behavior (Efrati and Mikulincer, 2018). In addition, problematic pornography use was the most common behavior reported by individuals seeking treatment for hypersexuality (Reid et al., 2012a, b). In a previous study, one in seven pornography users who participated in the survey indicated an interest in seeking treatment for their pornography use (Kraus et al., 2016a). However, most studies in the field have been conducted in Western countries (e.g., Grubbs et al., 2019a). In fact, 47.2% of previous studies were conducted using American samples, while only 7.9% were conducted in countries in the Global South (e.g., Brazil, China). Thus, there is a dearth of research among non-Western countries (Grubbs et al., 2020). Cross-cultural studies are needed to assess internet sex addiction, including problematic pornography use, as differences in sociocultural contexts may influence sexual behaviors (Griffiths, 2012). Moreover, data from the most popular pornography website worldwide^1^ revealed that Japan was ranked second in terms of daily traffic, only after the United States in the year 2019. Furthermore, online pornography use has been correlated with internet addiction scores in a sample from the national population of internet users in Japan (Yong et al., 2017). Thus, some individuals may report negative effects of pornography use due to loss of control as a result of internet addiction in Japan. However, there has been no research on either the risk of pornography addiction in Japan or the characteristics of Japanese pornography users. Additionally, although this concept has not yet been empirically validated, sexual desire and pornography may be considered a sensitive theme in the Japanese cultural context (Hirayama, 2019). In Japan, comprehensive sexuality education is not actively taught, so there are few opportunities to acquire basic knowledge regarding sexuality (Hashimoto et al., 2012). Public discussion of sexual desire and pornography is likely to cause embarrassment, and sexuality remains a taboo subject in Japan (Inose, 2010; Hirayama, 2019). In general, it has been noted that within the Japanese cultural context, issues of sexuality are a sensitive theme even in the academic field (Hirayama, 2019). Nonetheless, as mentioned earlier, Japanese people use a lot of pornography (see text footnote 1). Therefore, even if an individual in Japan is adversely affected by problematic pornography use, they may have difficulty seeking help and their behavior may not be recognized by clinicians. We believe it is necessary to conduct a study that focuses on the problematic use of pornography in Japan.

Previous studies have estimated the prevalence of addicted pornography use, despite difficulty determining the accurate prevalence of problematic pornography use in the general population using different assessment tools. For example, Rissel et al. (2017) found that 4% of men and 1% of women felt they were addicted to pornography. In another study, Bőthe et al. (2018) reported that there were almost 4% at-risk pornography users in the sample. Concerning general internet addiction, if not pornography, the prevalence of severe addiction among Japanese adults was 6.1% for men and 1.8% for women (Lu et al., 2011). In addition, frequent internet usage and negative mood states predict problematic online pornography use in young males such as adolescents (de Alarcón et al., 2019). Sexual behaviors related to impaired control are more common among men as compared to women (Kafka, 2010; Reid et al., 2012b; Kraus et al., 2016b). It is not clear whether problematic pornography users in Japan, who use pornography excessively and experience negative mood states, are more common among men rather than young women.

Problematic pornography use is associated with general psychopathological symptoms (Brand et al., 2011; Grubbs et al., 2015). It is also associated with poor psychosocial functioning, such as low satisfaction with life and relationships, among university students (Harper and Hodgins, 2016). In particular, individuals who engaged in problematic high-frequency pornography use reported high levels of hypersexuality and depression (Bőthe et al., 2020). Motivation for pornography use is based on an attempt to escape from negative feelings correlated with anxiety, loneliness, impulsiveness, and depression (Reid et al., 2011; Baltieri et al., 2015). In addition, the Interaction of Person-Affect-Cognition-Execution (I-PACE) for the processes underlying the development and maintenance of behavioral addiction include inhibitory control and executive functioning as main components (Brand et al., 2016, 2019b). Given the level of general inhibitory control over executive functioning, moderate external or internal triggers relating to addictive behavior, and decisions to engage is limited (Brand et al., 2016, 2019b), problematic pornography users may have low levels of inhibitory control. In relationships research that temperament and compulsive sexual behavior, effortful control, which is a temperamental dimension and is similar to executive function, was related to higher compulsive sexual behavior (Efrati, 2018). In fact, adolescents who exhibited clinical compulsive sexual behavior did not utilize effortful control (Efrati and Dannon, 2018). Furthermore, effortful control is known to comprise three difference functions (Rothbart et al., 2000): attentional control is the ability to focus attention appropriately or shift attention, inhibitory control is the ability to control inappropriate responses, and activation control is the ability to perform an action despite a strong tendency to avoid it. However, the relationship between the three functions and problematic pornography use has not been examined.

The current study aimed to identify the characteristics of pornography use and of individuals who experience problematic pornography use among Japanese students. First, we examined the percentage of Japanese university students who viewed pornography, the frequency of use within the past month, and the duration of use. Previous studies indicated that 4% of the study samples were high-risk pornography users and 16.8% were low-risk users (Bőthe et al., 2018). Thus, we hypothesized that approximately 4% of participants in the current study would display problems in their lives due to excessive pornography use, and the proportion of uses of those with dysregulation would be approximately 16% with impaired control of pornography use. We also hypothesized that the rate of male pornography users with impaired control would be four times higher than that of women, based on previous research (Rissel et al., 2017).

Second, we examined differences between individuals with impaired control and without impaired control of pornography use, focusing on depression, anxiety, sexual compulsivity, and executive attention. We hypothesized that pornography users with impaired control would show high levels of psychopathological symptoms such as depression and anxiety, consistent with previous literature (Bőthe et al., 2020). Additionally, given that problematic pornography users display high sexual impulsivity and low executive functioning (Brand et al., 2016, 2019b), we expected to observe that hypersexuality, and low levels of executive attention when compared with pornography non-users and pornography users without impaired control.

## Materials and Methods

### Participants and Procedures

The study was conducted using two methods of convenience sampling among college students at a university in midland Japan. In the first method, the first author visited the classroom and distributed recruitment letters, which included instructions to access the online link to the questionnaires to 216 students. In the second method, we sent a link to the online questionnaires to 70 students via LINE, a messenger app. Prior to responding to the questionnaires, all participants received information about ethical considerations, sensitive questions, and the right to withdraw. Participants gave their consent to participate through a link using Google forms. Prior to the participants answering questions on pornography, the operational definition of pornography was provided: Pornography (1) creates or elicits sexual thoughts, feelings, or behaviors, and (2) contains explicit images or descriptions of sexual acts involving the genitals (e.g., vaginal or anal intercourse, oral sex, or masturbation) (Hald and Malamuth, 2008; Reid et al., 2011). The response rate was 55.2% (*n* = 158). All research activities were approved by the institutional review board of (blinded for review).

Of the 158 respondents, some participants were excluded for being under 19 years of age (*n* = 3) or submitting incomplete questionnaires (*n* = 5). The final sample consisted of 150 Japanese students at a university in midland Japan (86 men; 57.3%, 64 females; 42.7%) aged 20–26 years (mean age = 21.48, *SD* = 1.21).

### Measures

#### Pornography Use

To assess the frequency of pornography use (number of days per month), we asked “How many days did you use pornography in the past month?” To assess the duration of use per day (in minutes), we asked “What is the average time you spend using pornography per day when you use it?”

#### Impaired Control of Pornography Use

To assess problematic pornography use, we used three yes/no questions: “Have you ever been unable to control overuse of pornography?”; “Have you repeatedly used pornography in an uncontrollable manner for more than 6 months?”; and “Have you faced daily-life problems due to difficulty in controlling pornography use?” These brief questions were developed by the authors of the present study who referred to the following proposed diagnostic criteria for compulsive sexual behavior disorder: failure to control intensity of sexual impulses or urges, repetitive sexual behaviors occurring over time, and significant impairment in one’s personal life (Kraus et al., 2018; World Health Organization, 2018). In particular, significant impairment in one’s personal life and impaired control of pornography use were important criteria from a clinical perspective (Harada, 2019).

#### Sexual Compulsivity

The Sexual Compulsivity Scale (SCS) has 10 items (e.g., “My sexual appetite has gotten in the way of my relationships”) rated on a four-point scale (1 = *not at all like me* to 4 = *very much like me*) with possible scores ranging from 10 to 40 (Kalichman and Rompa, 1995). High SCS scores indicate high-risk sexual behaviors (Kalichman and Rompa, 1995). In this study, the Japanese version of the SCS (Inoue et al., 2017) was used, which has verified internal consistency reliability (α = 0.90) and construct validity in Japan (Inoue et al., 2017). In the current sample, the reliability coefficient showed high internal consistency (α = 0.89).

#### Depression

The Patient Health Questionnaire (PHQ-9; Kroenke et al., 2001), a screening tool for major depression, has nine items (e.g., “Little interest or pleasure in doing things”) rated on a four-point scale (0 = *not at all* to 3 = *nearly every day*) with possible scores ranging from 0 to 27. In this study, the Japanese version of the PHQ-9 (Muramatsu et al., 2007) was used, which has verified reliability and validity. The reliability coefficient in previous studies was shown to be high (α = 0.86–0.92), and the PHQ-9 was found to be a valid measure of depression in the general population (Kroenke et al., 2010). In the current sample, the reliability coefficient showed high internal consistency (α = 0.81).

#### Anxiety

The seven-item Generalized Anxiety Disorder Scale (GAD-7; Spitzer et al., 2006) was used to assess anxiety symptoms, and the items (e.g., “Feeling nervous, anxious or on edge”) are measured on a four-point scale (0 = *not at all* to 3 = *nearly every day*) with possible scores ranging from 0 to 21. In this study, the Japanese version of the GAD-7 (Muramatsu et al., 2010) was used, which has exhibited good psychometric properties. The reliability coefficient (α = 0.92) and test-retest reliability (*r* = 0.83) were high in previous studies, and the scale was found to be a valid measure of generalized anxiety (Spitzer et al., 2006; Kroenke et al., 2010). In the current sample, the reliability coefficient showed high internal consistency (α = 0.86).

#### Effortful Control

The effortful control (EC) scale of the Adult Temperament Questionnaire (Rothbart et al., 2000) was used to measure executive attention function. The scale consists of the following three subscales, with a total of 35 items self-rated on a four-point scale (1 = *very false for me* to 4 = *very true for me*): attentional control (e.g., “It is very hard for me to focus my attention when I am distressed”) (12 items), inhibitory control (e.g., “I usually have trouble resisting my cravings for food, drinks, etc.”) (11 items), and activation control (e.g., “I can make myself work on a difficult task even when I don’t feel like trying”) (12 items). The total EC score was derived from the three subscale scores; high scores indicated high levels of EC. In this study, the Japanese version of the EC scale of the Adult Temperament Questionnaire (Yamagata et al., 2005) were used, which has verified reliability and validity. A previous study involving Japanese samples showed sufficient internal consistency (α = 0.74–0.90) and test-retest reliability *(r* = 0.79–0.89) for the scale, and criterion-related validity of relationships with five-factor personality dimensions (Yamagata et al., 2005). In the current sample, high α coefficients ranging from 0.72 to 0.88 demonstrated high reliability of the scale.

### Data Analysis

All statistical analyses were performed using IBM SPSS version 22. Prior to analyses, participants were categorized into three groups (pornography non-users, pornography users without impaired control, and pornography users with impaired control). To explore the association of sex, we used the Mann-Whitney *U* test and Pearson’s chi-square test. Thereafter, differences between the three groups were examined using non-normally distributed continuous variables (SCS, PHQ-9, and GAD-7 scores) with pairwise comparisons using Bonferroni adjustment, and one-way analysis of variance (ANOVA) for normally distributed continuous variables (EC total score and three subscale scores) with pairwise comparisons using a Tukey honestly significant difference adjustment.

## Results

### Pornography Usage Pattern

Participants reporting zero days of pornography use in the past month constituted pornography non-users (*n* = 44), those reporting use of pornography without a single “yes” to the impaired control questions constituted pornography users without impaired control (*n* = 81), and those reporting use of pornography with one or more “yes” responses to the impaired control questions constituted pornography users with impaired control (*n* = 25).

Considering all pornography users (*n* = 106) comprising those with and without impaired control, the mean frequency of use (in days) in the past month was 12.11 (*SD* = 8.21, min = 1, max = 31, skewness = 0.75, kurtosis = −0.19), and the duration of use (in minutes per day) was 44.60 (*SD* = 30.48, min = 1, max = 150, skewness = 1.45, kurtosis = 1.78). Additionally, pornography users with impaired control indicated a higher frequency of use than those without impaired control (*U* = 505.5, *p* < 0.001, *r* = 0.37); no significant difference was found between groups with respect to duration of use (*U* = 932.00, *p* = 0.541, *r* = 0.06). About one-fifth of pornography users with impaired control (*n* = 5) responded with a “yes” to all questions related to impaired control (Table 1).

**TABLE 1:** Comparison between pornography users without and with impaired control.

	Pornography users without impaired control	Pornography users with impaired control	
			
	(*n* = 81)	(*n* = 25)	
	*n* (%)/*Mean* (*SD*)	*n* (%)/*Mean* (*SD*)	*U*
**Frequency of pornography use**			
*Mean* (*SD*)	10.47 (7.64)	17.44 (7.85)	505.50***
1–10 (days)	56 (69.13%)	6 (24%)	
11–20 (days)	18 (22.22%)	14 (56%)	
21–31 (days)	7 (8.64%)	5 (28%)	
**Duration of Pornography use**			
*Mean* (*SD*)	44.30 (31.38)	45.60 (27.93)	932.00
1–29 (min)	23 (28.4%)	6 (24%)	
30–59 (min)	31 (38.3%)	9 (36%)	
60–89 (min)	17 (21.0%)	8 (32%)	
90–150 (min)	10 (12.4%)	2 (8%)	
**Impaired control of pornography use**			
Unable to control (yes)		22 (88%)	
Repeated use for more than six months (yes)		17 (68%)	
Daily-life problems (yes)		6 (24%)	
Total score = 1		10 (40%)	
Total score = 2		10 (40%)	
Total score = 3		5 (20%)	

***p < 0.001.

### Sex Differences in Usage Patterns

The scores of men (*M* = 13.19, *SD* = 7.68) and women (*M* = 8.22, *SD* = 9.02) differed significantly in relation to frequency of use (*U* = 519.00, *r* = 0.33, *p* < 0.001), while we found no significant differences between men (*M* = 43.35, *SD* = 28.19) and women (*M* = 49.13, *SD* = 38.01) with respect to duration of use (*U* = 934.00, *r* = 0.02, *p* = 0.872). Moreover, significant differences were observed in the proportion of men and women in the three groups: pornography non-use, and pornography use with and without impaired control [χ^2^ (2) = 64.99, *p* < 0.001, Cramer’s *V* = 0.66; Table 2].

**TABLE 2:** Comparison between pornography non-users, pornography users without impaired control, and pornography users with impaired control.

	Overall	(a) Pornography non-users	(b) Pornography users without impaired control	(c) Pornography users with impaired control		
						
	(*N* = 150)	(*n* = 44)	(*n* = 81)	(*n* = 25)		
	*n* (%)/*Mean* (*SD*)	*n* (%)/*Mean* (*SD*)	*n* (%)/*Mean* (*SD*)	*n* (%)/*Mean* (*SD*)	χ^2^/*F*	*Post hoc* analyses
Sex					64.99***	
Male	86 (57.3%)	3 (6.8%)	63 (77.8%)	20 (80.0%)		
Female	64 (42.7%)	41 (93.2%)	18 (22.2%)	5 (20.0%)		
SCS	15.19 (5.65)	11.75 (2.77)	15.22 (4.69)	21.16 (7.22)	44.13***	(a) < (b), (a) < (c), (b) < (c)
PHQ-9	5.63 (4.22)	5.73 (3.55)	4.85 (4.02)	7.96 (5.17)	8.56*	(b) < (c)
GAD-7	4.13 (3.93)	4.05 (4.21)	3.26 (3.29)	7.08 (4.05)	17.36***	(b) < (c), (a) < (c)
EC	89.73 (14.56)	92.82 (16.32)	90.32 (13.23)	82.36 (13.44)	4.46*	(c) < (a), (c) < (b)
Inhibitory control	31.07 (5.07)	30.98 (4.98)	31.70 (4.64)	29.20 (6.2)	2.38	
Activation control	29.59 (6.82)	31.05 (7.72)	29.48 (6.42)	27.40 (6.01)	2.33	
Attentional control	29.06 (6.57)	30.80 (7.41)	29.14 (6.19)	25.76 (5.02)	4.94**	(c) < (a)

EC, Effortful Control scale; GAD-7, The 7-item Generalized Anxiety Disorder Scale; PHQ-9, Patient Health Questionnaire-9; SCS, Sexual Compulsivity Scale. ***p < 0.001, **p < 0.01, *p < 0.05.

### Differences Between Users of Pornography

Results of a Kruskal-Wallis test and one-way ANOVA for continuous variables showed significant differences between the groups with respect to sexual compulsivity (*p* < 0.001), depression (*p* = 0.014), anxiety (*p* < 0.001), EC (*p* = 0.013), and attentional control (*p* = 0.008). However, there were no group differences for inhibitory control *(p* = 0.096) and activation control (*p* = 0.100).

## Discussion

We examined pornography user characteristics and assessed differences between pornography non-users, users, and problematic users in a sample of university students in Japan. To the best of our knowledge, there are currently no studies on problematic pornography use in Japan, thus, the present study is novel from a cultural perspective.

The findings of the present study suggest the possibility of problematic pornography use among Japanese students. Data showed that 5.7% (*n* = 6) of users reported significant daily-life problems. This finding is consistent with prior research that reported the estimated prevalence of problematic pornography use (Ross et al., 2012; Rissel et al., 2017; Bőthe et al., 2018). In addition, pornography users with impaired control were 23.5% (*n* = 25) of users. The data indicated a high level of hypersexuality among pornography users with impaired control when compared with other groups. The most predominant behavior reported among men seeking treatment for hypersexuality is pornography consumption (Reid et al., 2012a, b). Thus, although the diagnosis does not explicitly state that the problematic pornography use is a subtype of compulsive sexual behavior disorder (Gola et al., 2020), in line consistent with previous research, problematic pornography use may be considered a prominent behavioral manifestations of compulsive sexual behavior disorder (Brand et al., 2019a). Moreover, the high number of users with impaired control suggests the possibility that a large number of people have tendencies related to problematic pornography use in Japan. Additional research is needed.

Men reported a greater frequency of use and were more likely to be identified as problematic users than women. These findings are consistent with several previous studies that reported greater pornography use among male participants, who were prone to problematic use (Harper and Hodgins, 2016). Considering that most of the pornography usage was reported by male participants, it may be concluded that pornography is widely used by male university students in Japan. In contrast, women demonstrated a low rate of pornography usage. Since women in Japan may mainly use comics as pornographic material (Mori, 2017), differences in the content of pornographic material may have contributed to the gender differences observed in the results of the present study. In addition, a recent review focused on women with compulsive sexual behavior indicated that severe compulsive sexual behavior and urges to engage in pornography use are lower among women than men (Kowalewska et al., 2020). However, there is a possibility of problematic use among women, as some women reported using pornography with impaired control in the present study. Given the general dearth of research on female pornography use (Kraus et al., 2016b; Kowalewska et al., 2020), there is a need for increased focus on this issue in the Japanese context, as well as on types of sexual content that women use in particular, and women’s patterns of sexual behavior.

This study indicates the specific characteristics of pornography users with impaired control. Frequency of use was significantly associated with problematic use, but duration of use was not. While other addictive behaviors focus on the time spent on the behavior, pornography use with masturbation will limit sexual stamina even if the pornography use is not problematic (Fernandez and Griffiths, 2019). Therefore, problematic pornography users may not spend much time in actual use. While some people may be able to control or regulate pornography use regardless of high frequency and duration of use (Brand et al., 2011; Kor et al., 2014; Grubbs et al., 2015; Bőthe et al., 2018), others may feel a loss of control in pornography use regardless of the duration of use.

Participants with impaired control displayed high levels of depression and anxiety. These findings are consistent with a previous study in which problematic pornography users showed psychopathological symptoms (Brand et al., 2011; Grubbs et al., 2015). Intrapsychic distress diagnosed in individuals with compulsive sexual behavior resulted from high levels of sexual interest and behavior (World Health Organization, 2018). In some individuals, psychological distress can arise due to perceived moral incongruence derived from religious beliefs related to pornography (Grubbs et al., 2019b). As many Japanese individuals are reportedly irreligious (Mandai et al., 2019), emotional distress related to pornography use in Japan may not be a result of religious belief. However, sexual desire is a taboo in the Japanese social context (Inose, 2010); therefore, it is possible that the incongruence between this taboo and actual behavior, such as pornography use, causes psychological distress.

The results of the present study showed that most men use pornography. In Japan, scientific research and social discourse on sexuality are taboo (Hirayama, 2019). It is forbidden for people under 18 years of age to use pornography, but this in itself is not scientifically or socially controversial (Hirayama, 2019). In fact, there is little comprehensive sex education offered in Japan (Hashimoto et al., 2012). However, it has been shown that many Japanese people, including adolescents, use pornography (see text footnote 1; The Japanese Association for Sex Education, 2019). This phenomenon may mean that many Japanese people are engaging in sexual behavior without having any knowledge about sexuality. Therefore, Japanese people may find it difficult to determine which sexual behaviors are problematic and which are not, because Japanese people are unable to discuss their sexual concerns, and they lack knowledge about sexuality (Hashimoto et al., 2012). Hence, future research focusing on sexuality and compulsive sexual behavior in Japanese culture may be needed.

Finally, the low score related to effortful control and attentional control may be associated with problematic pornography use. This result follows recent research showing that low levels of effortful control are associated with individual-based compulsive sexual behavior (Efrati, 2018; Efrati and Dannon, 2018). In addition, effortful control measures the efficiency of executive attention, which is similar to executive function. As low effortful control scores are associated with impulsive behavior (Meehan et al., 2013), this finding may be similar to a recent study that showed executive functions, such as inhibitory control and decision-making, may contribute to the development and progress of several types of addictive behaviors (Brand et al., 2019b). Results showed that impaired users of pornography indicated low levels of attentional control of the EC subscale, suggesting that dysfunctional attentional control may promote responses to pornography-related triggers. In a previous study, inhibitory control of the EC subscale was related to risky sexual behavior in older adolescents (Lafreniere et al., 2013). Thus, among the three functions of effortful control, there may be a difference in that the individual-based compulsive sexual behavior is associated with attentional control, and the partner-based behavior is associated with inhibitory control. To address this mechanism, executive function and effortful control need to be studied in more detail.

Despite its novelty and strengths, this study has some limitations. First, our data were cross-sectional, and causality of the results cannot be determined. Second, because we used convenience sampling among university students at a university in midland Japan, our results cannot be generalized to the Japanese population. Third, the sample size was relatively small, and may not permit generalizability of these findings to all Japanese university students. Moreover, questionnaires used in this study included a sensitive topic that focuses on pornography use, and participants were contacted by the first author, which might have limited accurate responses by reducing anonymity. Finally, impaired control of pornography use was measured through self-report questionnaires generated for this study. There has been a recent increase in studies developing validity tools for problematic pornography use (Fernandez and Griffiths, 2019). Thus, future research should be conducted with a varied sample using validated measures of problematic pornography use.

To the best of our knowledge, this is the first study on problematic pornography use in Japan. The findings suggest a possible risk of problematic pornography use in Japan. Men showed higher frequency of use and were more prone to impaired control than women. Individuals with impaired control showed high sexual compulsivity, depression, anxiety, and low effortful control. Further research should explore a varied Japanese sample using validated measures.

## Data Availability Statement

The raw data supporting the conclusions of this article will be made available by the authors, without undue reservation.

## Ethics Statement

The studies involving human participants were reviewed and approved by the institutional review board of the faculty of education at Shinshu University. The patients/participants provided their written informed consent to participate in this study.

## Author Contributions

YO collected the data, conducted the statistical analysis, and wrote the first draft of the manuscript. YO, FT, and DI contributed to and approved the final manuscript. All authors contributed to the conceptualization of the research.

## Conflict of Interest

The authors declare that the research was conducted in the absence of any commercial or financial relationships that could be construed as a potential conflict of interest.

## Footnotes

1 PornHub.com
